# Potential mediators of *in ovo* delivered double stranded (ds) RNA-induced innate response against low pathogenic avian influenza virus infection

**DOI:** 10.1186/s12985-018-0954-2

**Published:** 2018-03-12

**Authors:** Hanaa Ahmed-Hassan, Mohamed Sarjoon Abdul-Cader, Upasama De Silva Senapathi, Maha Ahmed Sabry, Eman Hamza, Eva Nagy, Shayan Sharif, Mohamed Faizal Abdul-Careem

**Affiliations:** 1 Department of Ecosystem and Public Health, University of Calgary, Health Research Innovation Center 2C53, 3330 Hospital Drive NW, Calgary, AB T2N 4N1 Canada; 2 Department of Pathobiology, University of Guelph, Guelph, ON N1G 2W1 Canada; 3 Zoonoses Department, Faculty of Veterinary Medicine, Cairo University, Giza, 12211 Egypt

**Keywords:** *In ovo*, dsRNA, Type I interferon, Macrophage, Low pathogenic avian influenza virus, Chicken

## Abstract

**Background:**

Toll like receptor (TLR) 3 is a critically important innate pattern recognizing receptor that senses many viral infections. Although, it has been shown that double stranded (ds) RNA can be used for the stimulation of TLR3 signaling pathway in a number of host-viral infection models, it’s effectiveness as an antiviral agent against low pathogenic avian influenza virus (LPAIV) needs further investigation.

**Methods:**

In this study, first, we delivered TLR3 ligand, dsRNA, *in ovo* at embryo day (ED)18 since *in ovo* route is routinely used for vaccination against poultry viral and parasitic infections and infected with H4N6 LPAIV 24-h post-treatment. A subset of *in ovo* dsRNA treated and control groups were observed for the expressions of TLR3 and type I interferon (IFN)s, mRNA expression of interleukin (IL)-1β and macrophage recruitment coinciding with the time of H4N6 LPAIV infection (24 h post-treatment). Additionally, Day 1 chickens were given dsRNA intra-tracheally along with a control group and a subset of chickens were infected with H4N6 LPAIV 24-h post-treatment whereas the rest of the animals were observed for macrophage and type 1 IFN responses coinciding with the time of viral infection.

**Results:**

Our results demonstrate that the pre-hatch treatment of eggs with dsRNA reduces H4N6 replication in lungs. Further studies revealed that *in ovo* delivery of dsRNA increases TLR3 expression, type I IFN production and number of macrophages in addition to mRNA expression of IL-1β in lung 24-h post-treatment. The same level of induction of innate response was not evident in the spleen. Moreover, we discovered that dsRNA elicits antiviral response against LPAIV correlating with type I IFN activity in macrophages in vitro. Post-hatch, we found no difference in H4N6 LPAIV genome loads between dsRNA treated and control chickens although we observed higher macrophage recruitment and IFN-β response coinciding with the time of viral infection.

**Conclusions:**

Our findings imply that the TLR3 ligand, dsRNA has antiviral activity *in ovo* and in vitro but not in chickens post-hatch and dsRNA-mediated innate host response is characterized by macrophage recruitment and expressions of TLR3 and type 1 IFNs.

## Background

Avian influenza virus (AIV) infection is considered an economically important viral infection in poultry industry and a serious threat to public health [1, 2]. AIV infected poultry can act as a source of virus for human infections depending on the subtype of AIV [3, 4]. Although number of AIV control measures including enhanced biosecurity measures, surveillance, stamping out and quarantine of infected and contact animals have been practiced, AIV infections in poultry is an increasing concern [5, 6, 7]. Recently, vaccination directed against certain subtypes of AIV has been  introduced [7]. Since vaccination may prevent the clinical disease but not the infection, generation of diverse subtypes has become an additional concern [8–10]. Therefore, investigating new alternative and complementary strategies targeting AIV is a necessity [11].

One of the control options gaining increasing attention is the induction of broadly effective innate host responses. The generation of innate host responses is based on recognizing highly conserved pathogen-associated molecular patterns (PAMPs) that are distinct from the host. As part of the innate immune system, host pattern recognition receptors (PRR) engage PAMPs such as nucleic acids derived from pathogens including bacteria and viruses [12]. Polyinosinic–polycytidylic acid [Poly(I:C)] is a synthetic double stranded (ds)RNA stretch and an analog of a PAMP generated during the replication of RNA and DNA viruses [13], and is recognized by distinct receptors depending on their localization within the host cell. Extracellular viral dsRNA or its synthetic analog poly(I:C) can be detected by toll-like receptor (TLR)3 [14] activating toll/interleukin 1 receptor (TIR) domain-containing adaptor inducing interferon (IFN)- β (TRIF) pathway leading to type 1 IFN production. On the other hand, the retinoic acid inducible gene I (RIG-I)-like receptors (RLRs) and melanoma differentiation-associated gene 5 (MDA5) detect intracellular dsRNA and signal through the adaptor molecule mitochondrial antiviral signaling protein (MAVS) ultimately leading to the activation of the transcription factors such as nuclear factor (NF)-κB and interferon regulatory factor (IRF) 3 subsequently producing type I IFN and pro-inflammatory cytokines [15].

Members of the type I IFN family elicit antiviral response by binding to a common receptor, interferon alpha receptor which is located on the cell membrane of most host cells [16–18]. Upon engagement of type I IFNs and their receptor, downstream signaling is initiated through the Janus kinase/signal transducers and activators of transcription (JAK-STAT) pathway, thus inducing the transcription of IFN- stimulated genes (ISGs) [19]. ISGs are responsible for their innate immune functions including antiviral response [20, 21].

In chickens, TLR3 signalling dependent antiviral response has been shown against Newcastle disease virus (NCDV) in vitro [22]. Marek’s disease virus (MDV) replication in vitro has also been shown to be reduced with dsRNA treatment [23, 24]. Intramuscular delivery of dsRNA in chickens has also been shown to decrease cloacal and oropharyngeal shedding of low pathogenic (LP)AIV [25]. However, given the routine use of pre-hatch vaccination via *in ovo* route in poultry [26], it is unknown whether *in ovo* delivery of dsRNA elicits antiviral response against avian viruses. We hypothesized that expressions of TLR3 and type I IFNs and macrophage recruitment will be  increased following *in ovo* or post-hatch delivery of dsRNA and infection with LPAIV, when these changes are observed in lungs, resulting in  decreased LPAIV replication. The objective of our study was to uncover the potential elements of *in ovo* or post-hatch delivered dsRNA-mediated induction of antiviral response against LPAIV. Our findings imply that dsRNA has antiviral activity against LPAIV when delivered *in ovo* coinciding with increased macrophage recruitment and expressions of TLR3 and type I IFNs in addition to increased mRNA expression of interleukin (IL)-1β in lung.

## Methods

### Animals

The specific pathogen free (SPF) eggs were bought from the Canadian Food Inspection Agency (CFIA), Ottawa, Canada and incubated at the Health Research Innovation Center (HRIC), University of Calgary in digital incubators (Rcom Pro 20 & 50, Kingsuromax 20 and Rcom MARU Deluxe max, Autoelex Co., Ltd., GimHae, GyeongNam, Korea).

### Virus, cells and TLR ligand

H4N6 LPAIV, A/Duck/Czech/56, which was kindly provided by Dr. Eva Nagy (University of Guelph, Canada), was used in our studies after propagating in embryonated chicken eggs at embryo day (ED)9–11. The vesicular stomatitis virus expressing green fluorescent protein (VSV-GFP) and Vero cell line, a fibroblast-like kidney cell originated from African green monkey, were provided by Dr. Markus Czub (University of Calgary, Canada). Vero cells were used in the VSV-GFP propagation and titration. Madin-Darby Canine Kidney (MDCK) cells used for titrating H4N6 LPAIV were purchased from American Type Culture Collection (Manassas, VA, USA) and cultured in Dulbecco’s Modified Eagle’s Medium (DMEM) supplemented with 10% fetal bovine serum, penicillin (100 units/ml) and streptomycin (100 μg/ml) in a 5% CO_2_ incubator at 37 °C. MQ-NCSU (Muquarrab Qureshi-North Carolina State University) macrophage cell line was generously provided by Dr. Shayan Sharif, University of Guelph, Canada [27]. The MQ-NCSU cell line was maintained in LM-HAHN media which was prepared from 1:1 combination of McCoy’s 5A medium (Gibco, Life Technologies, Burlington, ON, Canada) and Leibovitz L-15 medium (Gibco, Life Technologies, Burlington, ON, Canada) supplemented with chicken serum (10%), fetal bovine serum (8%), 1 mM of 2-mercaptoethanol, sodium pyruvate (1%), L-glutamine (1%), penicillin (100 units/ml), streptomycin (100 μg/ml), tryptose phosphate broth (1%) and fungizone (250 μg/ml) in a 5% CO_2_ incubator at 40 °C.

The ligand for TLR3, synthetic dsRNA analog, Poly(I:C) HMW VacciGrade™ was purchased from InvivoGen (San Diego, California, USA) and reconstituted according to the manufacturer’s instructions.

### Experimental design

#### In vivo studies

##### Determination whether *in ovo* delivery of dsRNA induces antiviral response against H4N6 LPAIV infection

To determine whether *in ovo* delivery of dsRNA induces antiviral response against H_4_N_6_ LPAIV infection, SPF eggs at ED18 were candled to detect the fertility and viability of the embryos, the egg shell was disinfected with 70% ethanol and *in ovo* delivered [28] 250 μg dsRNA diluted in 200 μl of sterile PBS per egg (*n* = 3–5) or 200 μl of sterile PBS per egg (*n* = 3–4). The holes in the egg shell were sealed with lacquer and the eggs were incubated. After 24 h (ED19), both groups of eggs were infected with H_4_N_6_ LPAIV at 1X104 plaque forming units (PFU)/egg via *in ovo* route. At 1 day post-infection (ED 20), all the embryos were euthanized and the lungs were harvested in 1 ml of sterile PBS. For viral titration, the lungs that collected in 1 ml of sterile PBS were homogenized using Bio-Gen PRO200 homogenizer (Pro Scientific, Oxford, CT, USA), lung homogenate centrifuged at 3000 RPM, 4 °C for 10 min, aliquoted and stored at − 80 °C until used in plaque assay.

The plaque assay was done for titration of H4N6 LPAIV using MDCK cells. The MDCK cells were seeded in 6-well plates at 1 × 10^6^ cells per well concentration. After 24 h, the confluent monolayer of MDCK cells were washed twice with HBSS and inoculated with 10-fold serial dilutions of lung homogenates made in PBS. After 1 h of incubation at 37 °C, the plates were overlaid with serum-free 2X MEM media containing equal volume of 2.4% avicel® (FMC BioPolymer, Philadelphia, PA, USA) and 1 μg/ml of bovine TPCK-trypsin (SIGMA, Saint Louis, Missouri, USA). The inoculated plates were incubated for two days at 37 °C and 5% CO_2._ The visible plaques were counted under an inverted microscope after staining with 1% crystal violet.

##### Determination of the expressions of TLR3, type 1 IFNs and macrophage recruitment following *in ovo* delivery of dsRNA

To determine the mediators of antiviral response mediated by *in ovo* delivery of dsRNA, we delivered 250 μg dsRNA diluted in 200 μl of sterile PBS per egg or 200 μl of sterile PBS per egg (*n* = 4–6 per group) *in ovo* (ED18). Coinciding with the H4N6 LPAIV infection time, at ED19, the embryos were euthanized, the lungs and spleens were collected and preserved in optimum cutting temperature (OCT) compound (VWR International, Mississauga, ON, Canada) or RNA Save (Biological Industries, Cromwell, CT, USA). The sections (5 μ thickness) were made from frozen tissue blocks and immunofluorescence assays were performed to quantify the expressions of TLR3, IFN-α and IFN-β and macrophage recruitment in lungs. In the spleen, the expressions of IFN-α and IFN-β and macrophage recruitment were quantified. In addition, we did RT-PCR assay for the quantification of mRNA expression of IL-1β in lungs.

##### Determination whether intra-tracheal delivery of dsRNA induces macrophage and type 1 IFN responses in chickens leading to antiviral response against H4N6 LPAIV infection

To determine whether intra-tracheal delivery of dsRNA induces antiviral response against H4N6  LPAIV infection, the day old chickens were treated with dsRNA (*n* = 4) at 250μg per egg or PBS (*n* = 5) and infected with H4N6 LPAIV (1 × 10^6^ PFU per chicken) intra-tracheally 24 h post-treatment. At 24 h post-infection, the lungs were collected in RNA Save (Biological Industries, FroggaBio, Toronto ON, Canada) for the quantification of H4N6 LPAIV genome loads.

To determine whether intra-tracheally delivered dsRNA mediates expressions of type IFNs and macrophages recruitment in in lungs of chickens, another set of day old chickens were treated with dsRNA (*n* = 5) at 250μg per egg or PBS (*n* = 4) and lungs were collected in OCT for immunostaining of IFN-α, IFN-β and macrophages 24 h post-treatment coinciding with the intra-tracheal H4N6 LPAIV infection.

##### Immunofluorescence staining for the expression of TLR3, IFN-α, IFN-β and recruitment of macrophages

The frozen sections were brought to room temperature and fixed in ice cold acetone for 5 min. The sections were blocked with 5% horse serum for 1 h at room temperature and treated with primary antibodies; rabbit anti-chicken TLR3 (Creative diagnostics, New York, USA), rabbit anti-chicken IFN-β (Bio-Rad Laboratories, Mississauga, ON, Canada), rabbit anti-chicken IFN-α (Bio-Rad Laboratories, Mississauga, ON, Canada) or mouse monoclonal antibody specific for chicken macrophages, KUL01 (Southern Biotech, Birmingham, Alabama, USA) at dilutions of 1:50–200 in the blocking buffer for 30–60 min at room temperature in a humidified chamber. As the secondary antibody for TLR3, IFN-α and IFN-β, VectaFluor™ Excel Amplified DyLight® 488 Anti-Rabbit IgG Kit (Vector Laboratories, ON, Canada) and, as the secondary antibody for macrophage staining, DyLight® 550 conjugated goat anti-mouse IgG (H + L) (Bethyl Laboratories Inc., Montgomery, TX, USA) were used following the manufacture’s instruction. Nuclear staining was done using Vectashield mounting medium with DAPI (Vector Laboratories Inc., Burlingame, CA, USA), all the slides were cover slipped and sealed with lacquer.

##### RNA extraction and real time reverse transcription polymerase chain reaction (RT-PCR assay) for the quantification of IL-1β mRNA and H4N6 LPAIV genome load in lungs

Total lung RNA extraction was done using Trizol® reagent (Invitrogen Canada Inc., Burlington, ON, Canada) following the manufacturer’s instructions [29]. The Nanodrop 1000 spectrophotometer (ThermoScientific, Wilmington, DE, USA) was used to measure the concentration of the RNA at the wavelength of 260 nm. Five hundred nanograms (ng) of the extracted RNA was used to generate complementary DNA (cDNA) using reverse transcription random primers (10X) from High Capacity cDNA Reverse Transcription Kit (Invitrogen Life Technologies, Carlsbad, CA, USA) as per manufacturer’s guidelines. The real time RT-PCR assay was conducted in a 96-well PCR plate (VWR, Edmonton, AB, Canada) to quantify the mRNA expression of IL-1β in relation to β actin housekeeping gene. The numbers of copies of H4N6 LPAIV genome were normalized with β actin copy numbers. Fast SYBR^®^ Green Master Mix (Invitrogen, Burlington, ON, Canada) was used in 20 μl of reaction volume. The detection of intercalating SYBR^®^ Green dye was conducted in a Thermal Cycler (CFX96-C1000) (Bio-Rad Laboratories, Mississauga, ON, Canada). Five picomolar (pM) of IL-1β gene specific primers (F: 5′- GTG AGG CTC AAC ATT GCG CTG TA -3′ and R: 5′- TGT CCA GGC GGT AGA AGA TGA AG -3′) [30], H4N6 LPAIV matrix (M)1 gene specific primers (F: 5’-TTC TAA CCG AGG TCG AAA CG-3′ and R: 5’-ACA AAG CGT CTA CGC TGC AG -3′) [31]or β actin primers (F: 5’-CAA CAC AGT GCT GTC TGG TGG TA-3′ and R: 5’-ATC GTA CTC CTG CTT GCT GAT CC -3′) [30] were used in each reaction and gene specific plasmids were included as a positive control and RNAse free water was included as a negative control. The optimum parameters for thermal cycling were 95 °C for 20 s (s) of pre-incubation, 95 °C for 3 s of 40 amplification cycles with the final segment of 60 °C for 30 s. Melting curve analysis was done at 95 °C for 10 s, 65 °C for 5 s and finally 9 °C for 5 s. Acquisition of fluorescent signals was performed at 60 °C for 30 s.

#### In vitro studies

##### Determination whether antiviral response against H4N6 LPAIV infection and type I IFN activity are elicited in avian macrophages following treatment with dsRNA

To determine antiviral response elicited by dsRNA in avian macrophages, MQ-NCSU cells were cultured in 6- well plates at a viable cell density of 1.5 × 10^6^ cells/well for 24 h and treated with dsRNA in RPMI 1640 medium (Gibco Life Technologies, Burlington, ON, Canada) at a concentration of 50 μg/ml [32, 33] or same media as the control. The plates were incubated for 24 h in a 5% CO_2_ incubator at 40 °C. All the cells were infected with H4N6 LPAIV at an multiplicity of infection (MOI) of 0.1 for 30 min, fresh RPMI 1640 medium were added to the cells and were incubated for further 24 h in a 5% CO_2_ incubator at 40 °C. The supernatants originated from macrophages were collected and assayed on MDCK cell monolayers to determine the H4N6 LPAIV titers.

To determine type 1 IFN activity resulted from dsRNA of macrophages by VSV-GFP bioassay, MQ-NCSU cells were cultured in 12- well plates at a viable cell density of 7.5 × 10^5^ cells/well for 24 h and treated with dsRNA in RPMI 1640 medium (Gibco Life Technologies, Burlington, ON, Canada) at a concentration of 50 μg/ml or the same media as the control. The plates were incubated for 24 h in a 5% CO_2_ incubator at 40 °C. Then the cells were infected with VSV-GFP at an MOI of 0.1 for 30 min, fresh RPMI 1640 medium was added and incubated for further 24 h in a 5% CO_2_ incubator at 40 °C. We used VSV as a model pathogen, due to its high sensitivity to type I IFN activity, which has been well documented [34, 35]. The plates were washed twice with HBSS, fixed with 4% paraformaldehyde, washed again with HBSS and nuclear staining was done with Hoechst 33,342 (33,342 (Image-iT™ LIVE Plasma Membrane and Nuclear Labeling Kit (I34406), Invitrogen, Eugene, Oregon, USA) per the manufacturer’s instructions.

### Data analyses

#### Type I IFN activity analysis

To quantify the percentage of cells expressing VSV-GFP in type I IFN bio assay, the plates were scanned using the In-Cell Analyzer 2000 (GE Healthcare Sciences, Mississauga, Ontario, Canada) and further analysis of images were done through multi-target analysis using In Cell Analyzer workstation 1000 software version 3.7 (GE Healthcare Sciences, Mississauga, Ontario, Canada).

#### Analysis of immunofluorescent stained cells

To quantify the positive signals of IFN-β and IFN-α staining, figures were captured (5 fields/well) using an epifluorescent microscope (Olympus IX51, Markham, Ontario, Canada). For the quantification of positive TLR3 staining, 24 fields/well were captured using the In Cell Analyzer 2000 (GE Healthcare Sciences, Mississauga, Ontario, Canada). The fluorescent intensity was measured using Image-J software (National Institute of Health, Bethesda, Maryland, USA).

#### Statistical analysis

The statistical analysis was done using Student’s *t*-test (GraphPad Prism Software 5, La Jolla, CA, USA) to identify differences between two groups. The differences between groups were considered significant at *p* ≤ 0.05.

## Results

### *In ovo* delivery of dsRNA reduces H4N6 LPAIV infection encountered pre-hatch

**Fig. 1:**
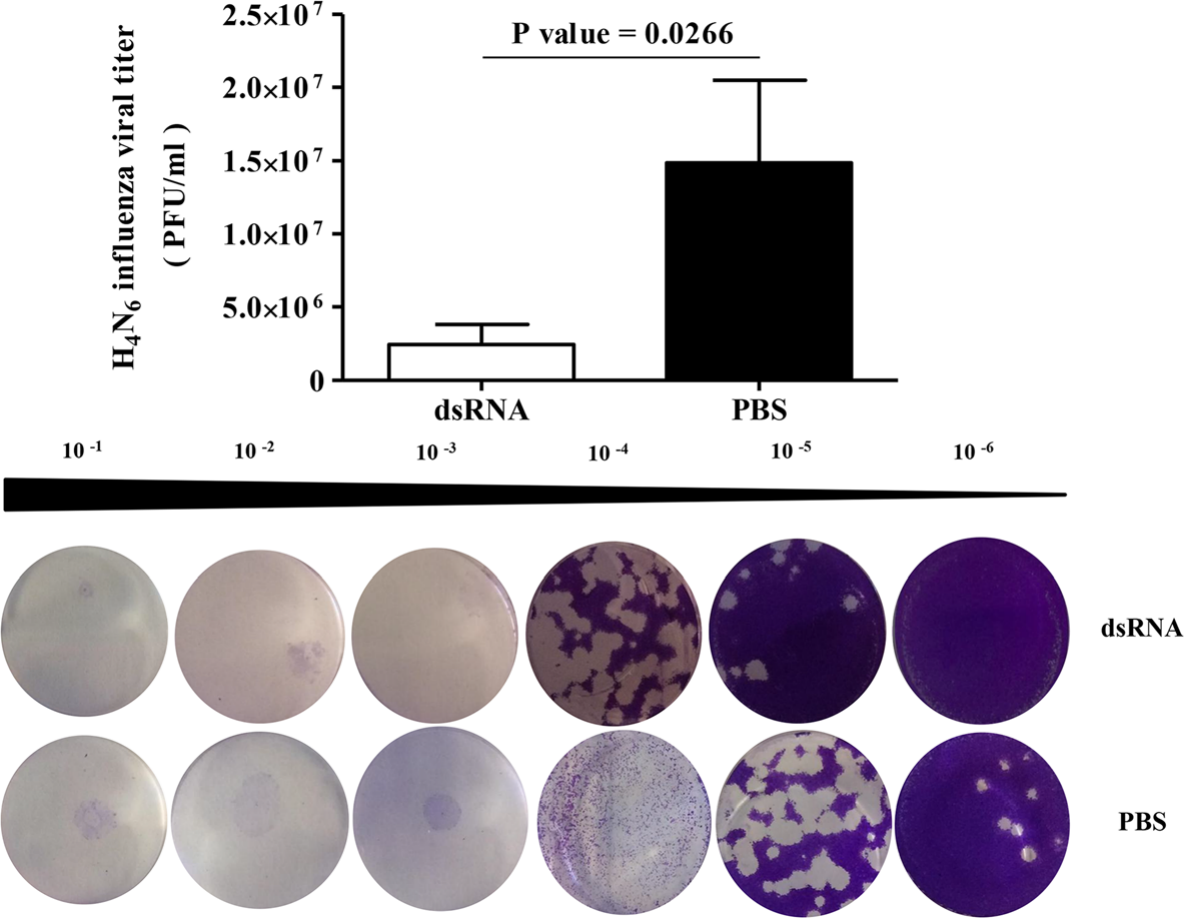
*In ovo* delivery of dsRNA reduces H4N6 LPAIV infection encountered pre-hatch**.** SPF ED18 eggs were treated either with dsRNA (*n* = 3–5) at 250μg per egg or PBS (*n* = 3–4) and infected with 1X10^4^ PFU per eggs H4N6 LPAIV at ED19 *in ovo*. The lungs were isolated in 1 ml PBS at ED20 and viral titers were determined on MDCK cells using plaque assay. Student’s *t*-test was done to identify group differences and the differences were considered significant at *P* < 0.05. The quantitative data are given as the mean ± standard error of mean (SEM) and representative images of plaque assay plates are also given for each group. The data represent pooled data of two independent experiments

H4N6 AIV titers in lungs of dsRNA treated and controls are illustrated in Fig. 1. We observed that the *in ovo* delivery of dsRNA at ED18 significantly reduces the H4N6 LPAIV replication in lungs pre-hatch when compared to controls that received PBS at ED18 (*P* = 0.0266).

### *In ovo* delivery of dsRNA induces innate host responses characterized by innate cell recruitment and the expressions of innate mediators in lungs and not in spleen

**Fig. 2:**
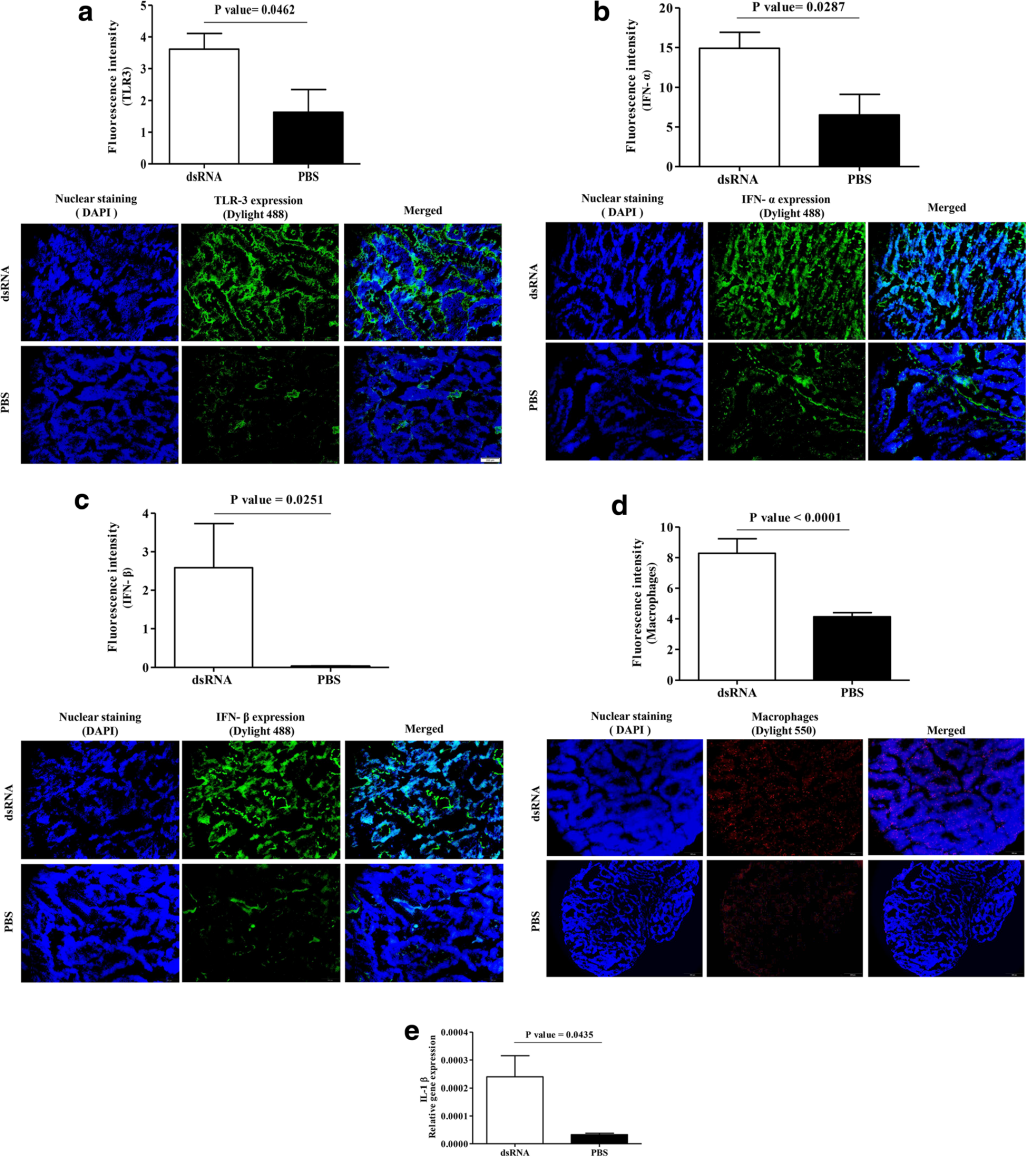
*In ovo* delivered dsRNA results macrophage recruitment and the expressions of innate immune mediators in lungs. SPF ED18 eggs were treated with dsRNA (*n* = 6) at 250μg per egg or PBS (*n* = 6) and, at ED19 coinciding with time of infection with H4N6 LPAIV, the lungs were collected in OCT for immunostaining of (**a**) TLR3 (**b**) IFN-α (**c**) IFN-β and (**d**) macrophages. Real time PCR assay was done in lungs to quantify mRNA expression of IL-1β (**e**) Student’s *t*-test was done to identify group differences and the differences were considered significant at *P* < 0.05. The quantitative data are given as the mean ± SEM. The representative immunoassayed images of nuclear stained, antigen stained and merged pictures are also given for each group and for each examined protein

Since *in ovo* delivered dsRNA reduces H4N6 LPAIV replication in lungs, first, we observed whether ED18 delivered dsRNA has increased the expression of TLR3 in lungs coinciding with the time of H4N6 AIV infection (ED19). We found that *in ovo* delivery of dsRNA has increased TLR3 expression in ED19 lungs when compared to the controls that received PBS (*P* = 0.0462, Fig. 2a). Second, we observed the expression of type I IFNs (namely IFN-α and IFN-β) in lungs at ED19, since these two molecules are produced downstream of TLR3 activation. As expected, we observed that *in ovo* delivery of dsRNA increased expressions of IFN-α (*P* = 0.0287, Fig. 2b) and IFN-β (*P* = 0.0251, Fig. 2c) in lungs 24 h post-treatment. Third. we determined whether the *in ovo* delivered dsRNA could recruit macrophages in lungs and we observed that *in ovo* delivered dsRNA significantly increased the number of macrophages in lungs at ED19 (*P* = 0.0001, Fig. 2d). Fourth, we observed that *in ovo* delivered dsRNA significantly increased the mRNA expression of IL-1β in lungs at ED19 (*P* = 0.0435, Fig. 2e).

**Fig. 3:**
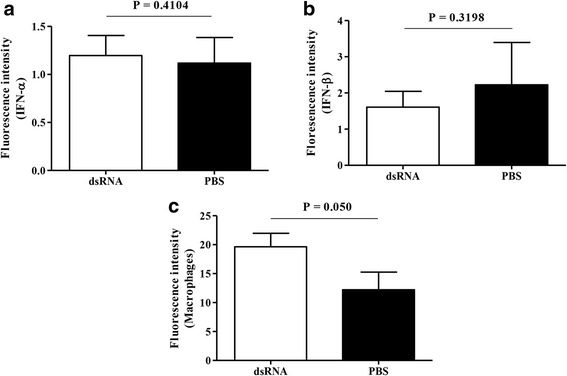
*In ovo* delivered dsRNA results only marginally higher macrophage recruitment in spleen. SPF ED18 eggs were treated with dsRNA (*n* = 4) at 250μg per egg or PBS (*n* = 4) and, at ED19 coinciding with time of infection with H4N6 LPAIV, the spleens were collected in OCT for immunostaining of (**a**) IFN-α (**b**) IFN-β and (**c**) macrophages. Student’s *t*-test was done to identify group differences and the differences were considered significant at *P* < 0.05. The quantitative data are given as the mean ± SEM

The expressions of IFN-α (*P* = 0.4104, Fig. 3a) and IFN-β (*P* = 0.3198, Fig. 3b) were not different in *in ovo* dsRNA delivered spleens when compared to that of the controls. However, macrophage numbers were marginally higher in *in ovo* dsRNA delivered spleen when compared to the controls (*P* = 0.05, Fig. 3c).

### Intra-tracheal delivery of dsRNA induces macrophage and IFN-β responses in lung of chickens without affecting H4N6 LPAIV infection

**Fig. 4:**
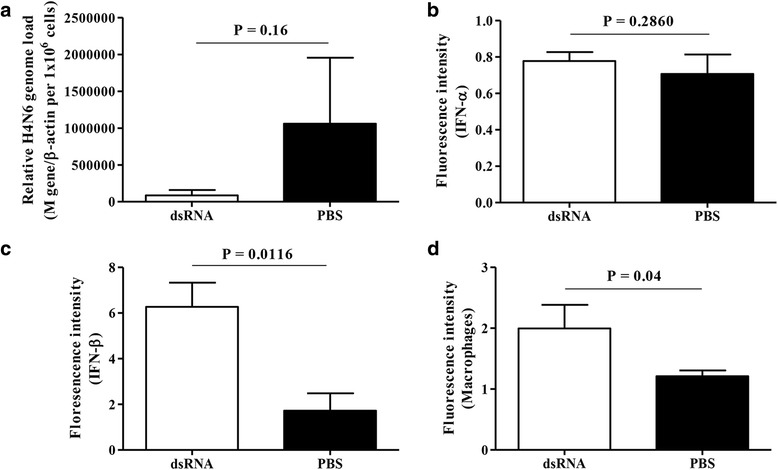
Intra-tracheal delivery of dsRNA in chickens results macrophage recruitment and the expression of IFN-β in lungs with no effect on H4N6 LPAIV infection. Day old chickens were treated with dsRNA (*n* = 4) at 250μg per egg or PBS (*n* = 5), infected with H4N6 LPAIV (1 × 10^6^ PFU per chicken) intra-tracheally 24 h post-treatment and the lungs were collected in RNA Save for the quantification of H4N6 LPAIV genome loads 24 h post-infection (**a**) Separately another set of day old chickens were treated with dsRNA (*n* = 5) at 250μg per egg or PBS (*n* = 4) and lungs were collected in OCT for immunostaining of (**b**) IFN-α (**c**) IFN-β and (**d**) macrophages 24 h post-treatment coinciding with the H4N6 LPAIV infection. Student’s *t*-test was done to identify group differences and the differences were considered significant at *P* < 0.05. The quantitative data are given as the mean ± SEM

H4N6 LPAIV genome loads in lungs of dsRNA treated and control chickens are illustrated in Fig. 4a. Unexpectedly, we observed that the intra-tracheal delivery of dsRNA at the day of age did not affect H4N6 LPAIV infection in lungs when compared to the controls that received PBS (*P* = 0.16).

The expression of IFN-α was not different in lungs of intra-tracheal dsRNA and PBS treated chickens (*P* = 0.2860, Fig. 4b). However, we observed that intra-tracheal delivery of dsRNA increased the expression of IFN-β (*P* = 0.0116, Fig. 4c) and recruitment of macrophages (*P* = 0.04, Fig. 4d) in lungs of chickens 24 h post-treatment.

### dsRNA treatment of avian macrophages elicits antiviral response against H4N6 LPAIV infection associating with type I IFN activity in vitro

**Fig. 5:**
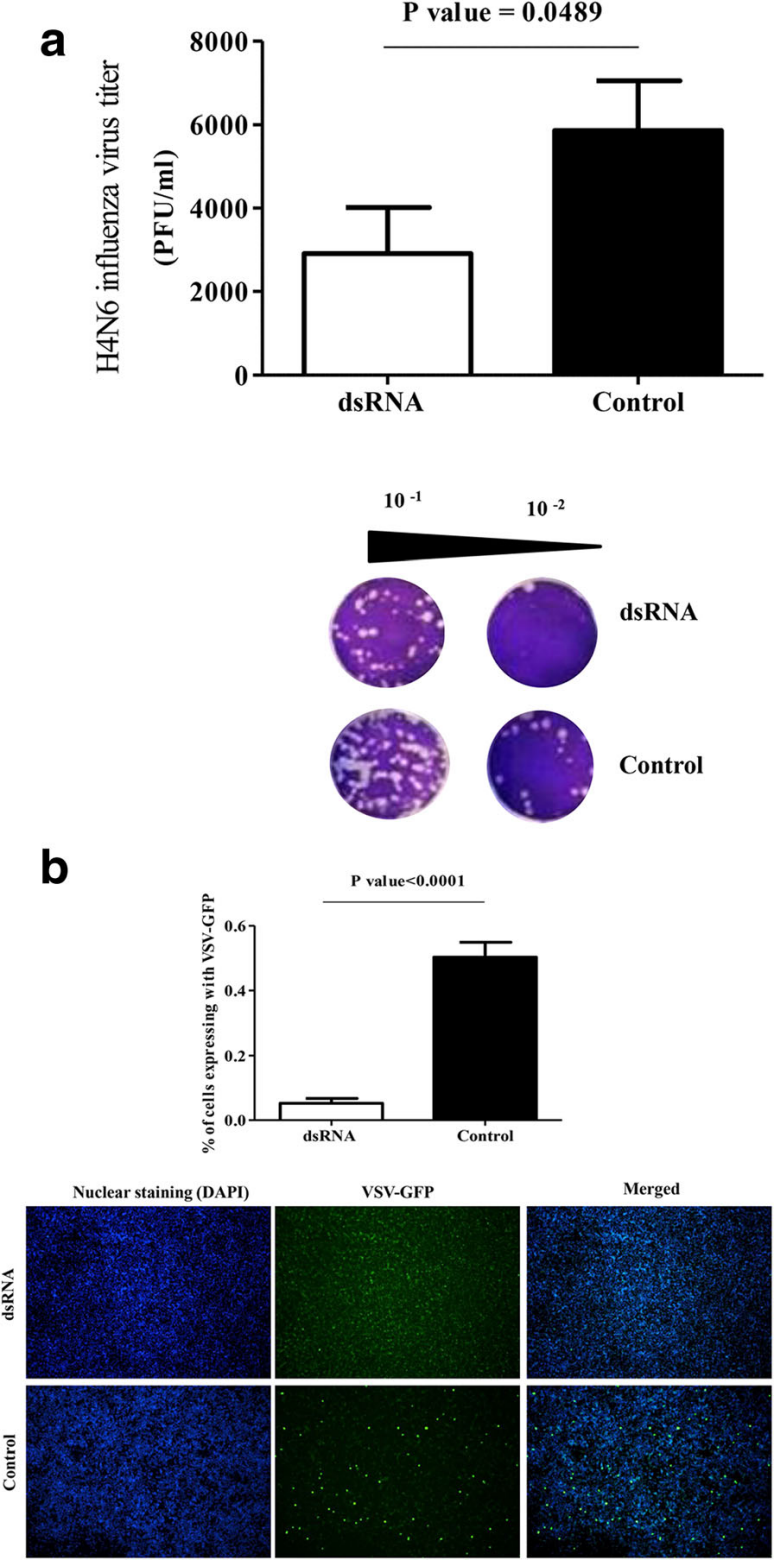
dsRNA treatment of macrophages elicits antiviral response against LPAIV infection and type I IFN activity**.** Avian macrophages were cultured in 6- well plates or 12- well plates at 1.5 × 10^6^ or 7.5 × 10^5^ cells per well respectively for 24 h and then, treated with dsRNA at 50 μg/ml (*n* = 3) or RPMI medium as control (*n* = 3) for 24 h. (**a**) MQ-NCSU cells infected with H4N6  LPAIV (MOI = 0.1) for 24 h, supernatants were collected and plaque assay was done on MDCK cells. (**b**) For type 1 IFN activity measurement, the MQ-NCSU cell monolayers were infected with VSV-GFP (MOI = 0.1) for 24 h and the plates were scanned by In Cell Analyzer 2000 machine for green fluorescent signals and further analyzed by In Cell Analyzer 1000 software. Student’s *t*-test was done to identify group differences and the differences were considered significant at *P* < 0.05. The data are expressed as the mean ± SEM and the results represents pooled data of 3 independent experiments. The representative images of nuclear stained, VSV-GFP positive and merged pictures are also given for each group

Since we observed that *in ovo* delivery of dsRNA treatment elicits innate response in lungs characterized by macrophage recruitment, we sought to investigate whether macrophages play a clear role in antiviral response against H4N6 LPAIV in vitro. We found that the treatment of avian macrophages with dsRNA at 50 μg/ml concentration for 24 h elicits antiviral response against H4N6 LPAIV (*P* = 0.0489, Fig. 5a). We also determined whether dsRNA treatment of macrophages leads to type I IFN activity in vitro*.* We found that when the macrophages were infected with H4N6 LPAIV, the type I IFN activity was significantly higher in dsRNA treated macrophages when compared to the controls (*P* = 0.0001, Fig. 5b).

## Discussion

*In ovo* route has been used routinely for the delivery of vaccines for the control of number of viral infections in chickens [28] ever since *in ovo* vaccination has been introduced for the control of Marek’s disease in chickens by Sharma and Burmester [36]. Induction of innate antiviral responses has also been shown using *in ovo* delivered cytosine-guanosine deoxynucleotides (CpG) DNA [37–40] which signal through TLR21 in chickens. In this context, the work described in the manuscript reveals a number of mechanisms relevant to *in ovo* delivered dsRNA-mediated innate host response elicited against LPAIV replication in avian species. First, we found that *in ovo* delivered dsRNA could elicit antiviral response reducing LPAIV replication in lungs pre-hatch. Second, we showed that dsRNA-mediated innate response correlates with the higher expressions of TLR3, type I IFNs, macrophage recruitment and mRNA expression of IL-1β in lungs pre-hatch. Third, we observed that although macrophages are marginally higher in the spleen, induction of type 1 IFN response was poor in this tissue pre-hatch. Fourth, we recorded that although dsRNA induced macrophages and IFN-β responses in chickens, it did not lead to antiviral response against H4N6 LPAIV in lungs post-hatch. Finally, we observed that macrophages, when treated with dsRNA, are capable of eliciting antiviral response against LPAIV correlating with type I IFN activity.

The results of in vivo studies in mammalian hosts suggest that activation of TLR3 signaling pathway by its ligand, dsRNA, provides protection against a number of viral infections. For example, dsRNA mediated antiviral response against herpes simplex virus (HSV) has been shown in a mouse model [41]. Further, intranasal delivery of dsRNA to mice has also been shown to provide protection against lethal challenge with H5N1, H1N1 and H3N2 influenza viral strains [42]. In chickens, parenteral administration of dsRNA has been shown to decrease LPAIV shedding [25]. Our findings imply that *in ov*o delivered dsRNA could elicit antiviral response reducing H4N6 LPAIV replication significantly in lungs pre-hatch although we observed that *in ovo* delivered dsRNA has not prevented H4N6 replication in lungs. This level of antiviral response corresponds to the level of innate host responses induced by dsRNA which was not substantial although significant statistically. However, when we delivered dsRNA post-hatch in chickens, the observed macrophage and IFN-β responses in lungs did not lead to antiviral response against H4N6 LPAIV infection in lungs similar to pre-hatch situation. This discrepancy in observations of antiviral responses between pre-hatch and post-hatch situations is difficult to explain. It is possible that the differences in routes of administrations (*in ovo* vs intratracheal) and the age of the animals (− 3 days vs + 1 day) may have contributed to the discrepancy although both antiviral responses were measured 24 h post treatment with same dsRNA dose (250 μg/ egg or chicken). In agreement with our view, it has been shown that pre-hatch lungs produce more type IFN activity when compared post-hatch lungs in response to viral infections [43].

In mammals, dsRNA is one of the TLR ligands that is potentially sensed by multiple innate receptors such as TLR3 [44], RIG I or MDA5 [45]. However, chickens rely on either TLR3 [46] or MDA5 [47] and not RIGI [48] for detecting dsRNA. Therefore, it is possible that the antiviral response we observed against H4N6 LPAIV may have been due to the dsRNA interaction with TLR3 and or MDA5. Of the two dsRNA recognizing receptors present in chickens, *in ovo* delivered dsRNA recognition potentially would have done by TLR3 rather than MDA5 due to following reasons. First, dsRNA treatment has been shown to alter the expression of mRNA of mammalian TLR3 in vitro [49–51]. In the current study, we also observed that the delivery of dsRNA *in ovo* increased the expression of TLR3 in lungs. Our observation agrees with other avian studies that showed increased mRNA expression of TLR3 gene in response to dsRNA treatment of chicken embryo fibroblast [52, 53]. Second, it is also important to note that MDA5 preferentially act as a receptor for short stretches of dsRNA strands [54] and, in our studies, we used long dsRNA strands as ligands for the induction of TLR3 signaling. Third, chicken MDA5 expression is not a critical factor that influences the antiviral response against AIV infection [47, 54].

In the mouse model, it has been shown that dsRNA mediated TLR3 activation lead to the recruitment of inflammatory cells in the respiratory tract [55] and agrees with our observation that dsRNA mediated macrophage recruitment to the lung pre- and post-hatch. Respiratory tract macrophages provide a first line of host defense against a range of airborne pathogens, including influenza virus by clearing infected and dying cells, secreting variety of cytokines and presenting antigen in order to elicit adaptive immune responses [56]. Furthermore as shown in mammals, respiratory tract macrophages may also play critical roles during influenza virus infection [57], specially minimizing the secondary bacterial infection [58]. Therefore, it is possible that the macrophage response we observed following *in ovo* dsRNA treatment may have contributed in reducing  the H4N6 LPAIV infection in lungs pre-hatch. This view is in agreement with our previous observation indicated that CpG DNA mediated recruitment of macrophages are involved in reducing H4N6 LPAIV replication [28].

We observed that activation of TLR3 signaling *via* *in ovo* delivered dsRNA leads to IFN-α and IFN-β production in lungs. In mammals, it is well known that TLR3 signaling leads to the production of IFN-α and IFN-β [59]. Although, there are no comparable work performed in chickens that demonstrated TLR3 signaling leads to IFN-α and IFN-β production in vivo, transcription of IFN-β gene following dsRNA treatment of avian fibroblast cells has been shown [46, 47]. Type I IFNs are effective in reducing avian viruses such as NCDV, infectious bursal disease virus, infectious bronchitis virus, MDV and influenza viral subtypes (H9N2, H1N1, H5N9) in vivo [60–64]. Although the time of H4N6 LPAIV infection coincides with higher expressions of innate mediators and recruitment of macropahges following *in ovo* delivery of dsRNA, whether these innate molecules and cells played roles in reducing H4N6 LPAIV replication need further investigation.

*In ovo* delivery of dsRNA, although has stimulated innate host responses characterised by macrophages and expression of type 1 IFNs, similar observations were not obsereved in spleen which is the main secondary lymphoid organ in chickens. We believe that this difference is related to the difference in distribution of dsRNA in spleen when compared to the lungs following *in ovo* delivery. It is known that *in ovo* delivered compounds distribute mainly in respiratory and gastrointestinal mucosa [65].

LPAIV infection in chickens impacts not only poultry industry but also other animal species including other livestock species and public health since poultry can be a major source of virus for transmission to these animal species. Due to the limitations in avian influenza control measures, novel control measures are necessary and that should be based on understanding of the mechanisms of host responses elicited against this virus. Our findings of dsRNA-mediated antiviral response against LPAIV attributable to type IFN activity and the expression of TLR3 are preliminary and need further investigations. Our study did not determine whether the dsRNA mediated induction of macrophage and type IFN activities are leading to antiviral response against other subtypes of LPAIV than H4N6 subtype. Although, induction of innate host responses is not isolated from adaptive immune responses, our study did not address whether dsRNA mediated innate responses lead to adaptive immune responses in chickens. Studies leading to clinical protection was also precluded in our studies, since, LPAIV infection models are known for lack of induction of clinical manifestations on its own [66].

## Conclusions

Our results demonstrate that the treatment of embryonated chicken eggs with dsRNA through *in ovo* route reduces H4N6 LPAIV replication in lungs pre-hatch and the potential correlates of innate host response mediated by *in ovo* delivered dsRNA includes higher type I IFN production, expressions of TLR3 and IL-1β mRNA and macrophage recruitment in lung. In the spleen, although marginally higher macrophages were observed type 1 IFN expressions were not observed following *in ovo* dsRNA delivery. Moreover, we discovered that the dsRNA reduces H4N6 LPAIV replication associating with increase of type I IFN activity in macrophages in vitro. However, an  antiviral response of dsRNA was not observed post-hatch although dsRNA treatment increased macrophage numbers and IFN-β response in lungs post-hatch. Our findings imply that the TLR3 ligand, dsRNA has antiviral activity *in ovo* and in vitro but not in chickens post-hatch.
